# Glutamatergic Neurotransmission Controls the Functional Lateralization of the mPFC in the Modulation of Anxiety Induced by Social Defeat Stress in Male Mice

**DOI:** 10.3389/fnbeh.2021.695735

**Published:** 2021-08-23

**Authors:** Nathália Santos-Costa, Daniela Baptista-de-Souza, Lucas Canto-de-Souza, Vinícius Fresca da Costa, Ricardo Luiz Nunes-de-Souza

**Affiliations:** ^1^Laboratory of Pharmacology, School of Pharmaceutical Sciences, Universidade Estadual Paulista, Araraquara, Brazil; ^2^Joint Graduate Program in Physiological Sciences (PIPGCF) UFSCar- Universidade Estadual Paulista, São Carlos, Brazil

**Keywords:** mPFC, anxiety, ΔFosB, CaMKII, glutamatergic neurotransmission, chronic social defeat stress, mice

## Abstract

The rodent medial prefrontal cortex (mPFC) is anatomically divided into cingulate (Cg1), prelimbic (PrL), and infralimbic (IL) subareas. The left and right mPFC (L and RmPFC) process emotional responses induced by stress-related stimuli, and LmPFC and RmPFC inhibition elicit anxiogenesis and anxiolysis, respectively. Here we sought to investigate (i) the mPFC functional laterality on social avoidance/anxiogenic-like behaviors in male mice subjected to chronic social defeat stress (SDS), (ii) the effects of left prelimbic (PrL) inhibition (with local injection of CoCl_2_) on the RmPFC glutamatergic neuronal activation pattern (immunofluorescence assay), and (iii) the effects of the dorsal right mPFC (Cg1 + PrL) NMDA receptor blockade (with local injection of AP7) on the anxiety induced by left dorsal mPFC inhibition in mice exposed to the elevated plus maze (EPM). Results showed that chronic SDS induced anxiogenic-like behaviors followed by the rise of ΔFosB labeling and by ΔFosB + CaMKII double-labeling bilaterally in the Cg1 and IL subareas of the mPFC. Chronic SDS also increased ΔFosB and by ΔFosB + CaMKII labeling only on the right PrL. Also, the left PrL inhibition increased cFos + CaMKII labeling in the contralateral PrL and IL. Moreover, anxiogenesis induced by the left PrL inhibition was blocked by NMDA receptor antagonist AP7 injected into the right PrL. These findings suggest the lateralized control of the glutamatergic neurotransmission in the modulation of emotional-like responses in mice subjected to chronic SDS.

## Introduction

Several studies have pointed out that ∼15% of adults in the world are affected by anxiety disorders, one of the most common psychiatric disorders (World Health Organisation, 2017; Alonso et al., 2018; Charlson et al., 2019). Stressful situations are a widely known predictor of anxiety conditions (Myin-Germeys et al., 2003). In this sense, interpersonal conflicts can promote a huge impact on emotional responses (Bolger et al., 1989; Nezlek and Plesko, 2001).

From a basic research perspective, Rodgers and Cole (1993) have published the first evidence about the anxiogenesis-like response in mice induced by physical confrontation with an aggressive male conspecific, a phenomenon known as social defeat stress (SDS). Posteriorly, the protocol was adapted to improve the expertise in evaluating affective-like disorders in rodents (Golden et al., 2011). Therefore, our group has used the SDS to investigate the neuronal basis of defensive behaviors in the attacked mouse (Costa et al., 2016; Faria et al., 2020; Victoriano et al., 2020).

Among the various limbic areas that modulate the emotional consequences induced by SDS (Cooper et al., 2015), the medial prefrontal cortex (mPFC) is a highlighted forebrain area for regulating the behavioral responses such as social avoidance induced by stress in mice (Diorio et al., 1993; Blanchard et al., 1998; Cerqueira et al., 2008). Furthermore, a body of evidence indicates that mPFC lesion alters the anxiety-related behavior in rats exposed to the elevated plus maze (EPM), a widely used animal model of anxiety (Rodgers and Johnson, 1995; Carobrez and Bertoglio, 2005), and social interaction test (Gonzalez et al., 2000; Lacroix et al., 2000; Shah and Treit, 2003), highlighting the relevance of this forebrain area as a potential target for the effects of anxiolytic drugs (e.g., McNaughton and Corr, 2004; Jaferi and Bhatnagar, 2007; Holmes and Wellman, 2009).

Previous studies have shown the aversive effects of various classes of drugs injected into the mPFC [i.e., blockade of β1 adrenergic (atenolol), muscarinic cholinergic (scopolamine), or ionotropic glutamatergic (ap-5) (Stern et al., 2010) receptors; excitotoxic lesion (Shah and Treit, 2003)]. Lisboa et al. (2010) showed that cobalt chloride [CoCl_2_, a synaptic inhibitor (Kretz, 1984) that does not interfere with the fibers of passage function (Lomber, 1999)] injections into the prelimbic (PrL) and/or infralimbic (IL) portions of the mPFC of rats induce anxiogenic- and anxiolytic-like effects, respectively, in rats exposed to innate (i.e., EPM and light–dark box) and learned (i.e., contextual fear conditioning and Vogel conflict) anxiety/fear tests. Besides the distinct aversive nature of the test used to evaluate anxiety-related responses, such discrepant findings could be related to the involvement of the mPFC subregions or even to the functional lateralization of this forebrain area (Sullivan and Gratton, 1999, 2002; Cerqueira et al., 2008; Lee et al., 2015; Costa et al., 2016; Faria et al., 2020; Victoriano et al., 2020), which were not taken into account in most studies investigating the role of the mPFC in the modulation of anxiety. Regarding functional lateralization, previous studies have demonstrated that inhibition of the left and right mPFC produces anxiogenic- and anxiolytic-like effects, respectively, in mice exposed to various tests of anxiety (Lee et al., 2015; Costa et al., 2016).

Besides the utilization of techniques of functional inhibition (for instance, with irreversible lesions or local injections of CoCl_2_), the use of immunofluorescence assays is also convenient to investigate the role of a brain area in the modulation of an emotional state. In this context, the protein Fos has probably been the most commonly used neuronal activity marker in behavioral research, including studies on fear and anxiety (e.g., Morgan and Curran, 1991; Brenhouse and Stellar, 2006; Cruz et al., 2015). For instance, increased expression of protein Fos has been identified in limbic areas of animals exposed to the EPM (e.g., Duncan et al., 1996; Linden et al., 2003; Sorregotti et al., 2018). Moreover, Pati et al. (2018) have demonstrated the increase in Fos and the involvement of CaMKII [Ca2+/calmodulin-dependent protein kinase II, an NMDAR signaling activation-related protein (De Koninck and Schulman, 1998; Sanhueza et al., 2011; Carvajal et al., 2016)], in the mPFC of rats exposed to the EPM and open field tests.

Given that glutamate neurotransmission is ubiquitous in the PFC (McKlveen et al., 2015) and the mPFC plays a lateralized function in the control of anxiety (Cerqueira et al., 2008; Lee et al., 2015; Costa et al., 2016; Victoriano et al., 2020), we hypothesized that the elevation of anxiogenic-like responses induced by SDS is modulated by the glutamatergic neurotransmission in the mPFC. Furthermore, we hypothesize that the increase in anxiety-like behavior can be a consequence of hemispheric lateralization disturbance in subregions of this forebrain area.

To test these hypotheses, we investigated the influence of SDS protocol in mice on (i) the avoidance behavior assessed in the social interaction and (ii) EPM tests, and (iii) ΔFosB, CaMKII, as well as ΔFosB+CaMKII labeling in neurons located in both hemispheres of the mPFC of mice. Furthermore, we evaluated the (iv) presence of projections from the left to the right mPFC, (v) whether the anxiogenesis induced by LmPFC inhibition leads to cFos, CaMKII, and cFos + CaMKII labeling in neurons located in the RmPFC, and (vi) the effect of intra-RmPFC injection of AP7 (an NMDA receptor antagonist) on the anxiogenic-like effect induced by CoCl_2_ injection into the LmPFC in mice exposed to the EPM.

## Materials and Methods

### Subjects

One hundred sixty-eight male Swiss–Webster mice (São Paulo State University—Unesp, SP, Brazil) of 5–6 weeks of age were used in this study. Mice were housed in groups of 10 per cage (size: 41 × 34 × 16 cm) and maintained under a normal 12-h light cycle (lights on at 7:00 a.m.) in a temperature-controlled environment (23 ± 2°C). Food and water were freely available except during the brief test periods. All mice were naive at the beginning of the experiments, and they were used once. Housing conditions and experimental procedures were approved by the Ethical Committee for Use of Animals of the School of Pharmaceutical Sciences/Unesp, which complies with Brazilian and international guidelines for animal use and welfare (CEP/FCF/CAr-UNESP: protocol number 22/2017). All behavioral tests were performed randomly between 10 a.m. and 4 p.m.

### Drugs

The following drugs were used: cobalt chloride (CoCl_2_—non-specific synaptic blocker, 1 mM/0.2 μl) and AP7 (2-amino-7phosphonoheptanoic acid—an NMDA glutamate receptor antagonist, 0.05 nmol/0.2 μl). The drugs were dissolved in 0.9% physiological saline solution. Doses were based on previous studies (Costa et al., 2016; Faria et al., 2016; Victoriano et al., 2020).

### Surgery and Neurotracer and Drug Microinjection

Mice were bi- or unilaterally implanted (for details, see the section “General Procedure”) with a 7-mm stainless steel guide cannula (26 gauge; Insight Equipamentos Científicos Ltd., Brazil) targeted to the PrL, under anesthesia induced by intraperitoneal injection of ketamine (100 mg/kg) plus xylazine (10 mg/kg). Stereotaxic coordinates (Paxinos and Franklin, 2001) for the PrL were 1.94 mm anterior to bregma, ±0.3 mm lateral to the midline for left and right hemispheres, and 1.9 mm ventral to the skull surface, with the guide cannulae in the vertical position. To increase the accuracy of the bilateral surgery, both cannulas were implanted simultaneously by using an adaptor (that holds two cannulas at the same time) attached to the stereotaxic arm. The position of the head of the mouse is an important limitation for the efficacy of the surgery. It has to be as flat as possible (e.g., the height of the bregma needs to be in the same plane as the lambda).

For the neurotracer infusion experiment, 0.1 μl of the anterograde non-transsynaptic neurotracer BDA (Dextran Amine-Texas Red^®^, Biotinylated; Vector Laboratories) (Kim et al., 2019) was microinjected into the left prelimbic area. The BDA infusion was performed through an infusion pump (Micro4 Microsyringe Pump) linked to a syringe (0.5 μl, Neuros Syringe, Model 7000.5 KH, 32 gauge) in a ratio of 0.02 μl/min (final volume 0.1 μl). At the end of the infusion, the needle remained within the area for an extra 5 min (Reiner et al., 2000; Vercelli et al., 2000) to avoid the reflux of BDA.

For experiments with drug microinjections, guide cannulae were fixed to the skull with dental acrylic and jeweler’s screws. A dummy cannula (33 gauge, stainless steel wire; Fishtex Industry and Commerce of Plastics Ltd.), inserted into each guide cannula, served to reduce the incidence of occlusion. Immediately after surgery, the animals received an intramuscular injection of penicillin-G benzathine (Pentabiotic^®^, 56.7 mg/kg in a 0.1-ml volume; Fort Dodge, Campinas, SP, Brazil) and a subcutaneous injection of the anti-inflammatory analgesic Banamine^®^ (3.5 mg/kg of flunixin meglumine, Intervet Schering-Plough, Rio de Janeiro, RJ, Brazil, in a volume of 0.3 ml). Five to seven days after surgery, solutions (see the section “Drugs”) were injected into the mPFC through microinjection units (33 gauge stainless steel cannula; Insight Equipamentos Científicos Ltda., Brazil), which extended 0.1 mm beyond the tip of the guide cannula. Each microinjection unit was attached to a 2-μl Hamilton microsyringe via polyethylene tubing (PE-10). The microinjection procedure consisted of gently restraining the animal, removing the dummy cannula, inserting the injection unit *in situ*, and proceeding with the microinjection over a 30-s period, after which, the needle was left for a further 30 s. The final volume delivered was 0.2 μl. The successful procedure was verified by monitoring the movement of a small air bubble in the PE-10 tubing.

### Social Defeat Stress

Chronic SDS is based on the conflict between conspecifics and consists of the interaction between an aggressor resident and an intruder mouse placed in the cage of the aggressor. This aggressive interaction triggers various behavioral, endocrine, and autonomic changes in the defeated animal. The test has been used for the study of stress-related disorders, i.e., depression, anxiety, and drug abuse (Keeney and Hogg, 1999; Björkqvist, 2001; Hammels et al., 2015). The resident (Swiss–Webster, 10-60 weeks old; 40-55 g), an animal that displays spontaneous aggressive behavior, is socially isolated in individual cages (28 × 17 × 12 cm) with separated ventilation for at least 4 weeks to intensify their aggressive behavior. During the confrontation, the intruder remains in the home cage of the aggressor for 5 min maximum or until it presents a submissive posture: elevation of the body on the hind legs, front legs extended toward the aggressor, head retracted, and ears arched, for 3 s (Miczek and O’Donnell, 1978; Miczek et al., 1982). The aim of removing the intruder immediately after it presents a submissive posture was to avoid excessive injuries caused by the resident attack. The non-aggressive group (non-defeated intruders) were exposed to a familiar non-aggressive conspecific for 5 min in a cage similar to the cage of the resident. The non-aggressive group consisted of familiar mice that were housed together at the moment they arrived at the local animal facility. The aggressive or non-aggressive interactions were carried out once a day for 10 consecutive days. For aggressive interactions, each subject was randomly exposed to distinct aggressors, and immediately after each daily interaction, the intruders were returned to their home cages. To select the aggressor animals (residents) and perform the SDS, we followed a protocol similar to previous studies (Golden et al., 2011; Costa et al., 2016; Faria et al., 2020; Victoriano et al., 2020).

### Social Interaction Test

One day after the last aggressive or non-aggressive interaction, mice were subjected to the social interaction test. The social interaction arena (42 × 42 × 15 cm) and the behavioral analysis were adapted from those described by Golden et al. (2011). Briefly, the social interaction test was composed of two phases of 150 s each, separated by an interval of 30 s, either with or without the target (a non-familiar male resident) placed into a wire-mesh cage (10 × 6 × 15 cm), called the interaction zone (IZ). In the first phase, each mouse (previously subjected to the SDS or non-aggressive interaction) was placed on the opposite side of the IZ of the open field faced to the empty wire-mesh cage (no target) and was allowed to explore the arena. At the end of the first phase, the mouse was removed from the arena and left undisturbed in a holding cage for 30 s. During this time, the empty wire-mesh cage was replaced by a wire-mesh cage with a non-familiar resident mouse (target) preselected as aggressive. The second phase started when the experimental mouse was placed again in the arena, in the same position as described for the first phase, except that it now faced the wire-mesh cage where the resident mouse was in (target). All sessions were recorded under red light illumination (50 lx on the floor of the arena) by a vertically mounted camera linked to a monitor. The exploration time (in seconds) of the IZ and corner zones (CZ) were recorded in the absence (no target) and presence of the target. The social avoidance behavior was also expressed as a social interaction ratio, which is the ratio of time a mouse spends in the IZ or CZ in the presence of a target compared with the absence of a target. Between subjects, the arena was thoroughly cleaned with 20% alcohol.

### Elevated Plus Maze and Behavioral Analysis

The basic EPM design comprised two open arms (30 × 5 × 0.25 cm) and two closed arms (30 × 5 × 15 cm), connected via a common central platform (5 × 5 cm). The apparatus was constructed from wood covered with Formica (floor) and transparent glass (clear walls) and was raised to a height of 38.5 cm above the floor level, as originally described by Lister (1987). After drug injection (see the section *“*Surgery and Neurotracer and Drug Microinjection” for details), each mouse was placed in an individual holding cage and then transported to the maze. Testing commenced by placing the subject on the central platform of the maze (facing an open arm), following which the experimenter immediately withdrew to an adjacent room. The test sessions were 5 min in duration, and between subjects, the maze was thoroughly cleaned with 20% alcohol. All experiments were performed under low luminosity (50 lx on the central platform of the EPM), during the light phase of the light–dark cycle. All sessions were recorded by a vertically mounted camera linked to a monitor. The EPM videos were scored by using software (X-plo-rat 2005, University of São Paulo) (Tejada et al., 2018). Behavioral parameters comprised conventional spatiotemporal measures: frequencies of closed-arm entries, open-arm entries, and the time (in seconds) spent in the open arm of the maze (entry = all four paws into an arm). These data were used to calculate the percentage of open-arm entries [(open/total) × 100] and percentage of open-arm time [(time open/300) × 100] (Rodgers and Johnson, 1995).

### Immunofluorescence

Mice were transcardially perfused with 30 ml of 1X phosphate-buffered saline (PBS) at pH 7.4, followed by 50 ml of fresh 4% PFA. The brain was dissected and transferred to a 30% sucrose solution in PBS for 48 h at 4°C. After the brains had submerged, the tissues were embedded in OCT and sectioned at 35-μm thickness on a cryostat. Sections were placed in serial order in a 12-well plate containing 0.1 M phosphate buffer (PB) with 0.01% sodium azide. Sections were washed three times in 0.1 M PB and then incubated in a blocking solution, containing 10% normal goat serum and 0.3% Triton-X 100 in 0.1 M PB, for 1 h at room temperature with gentle rocking. Sections were incubated overnight with the primary antibody previously diluted in a blocking solution. The primary antibodies used were rabbit-anti-FOS (EUA—1:1,000 working dilution; Cat. No. 5348: SER-32 D82C12, Cell Signaling Technology Inc. Danvers, MA, United States), anti-ΔFosB (1:1,000; Cat. No. EPR15905; Abcam) and anti-mouse CaMKII (EUA—1:1,000 working concentration; Cat. No. TH269517: 6G9; Thermo Fischer Scientific. Rockford, IL, United States). Sections were washed five times in 0.1 M PB and then incubated for 2 h at room temperature with a secondary antibody (1:1,000 each) diluted in blocking solution. The secondary antibodies used were anti-rabbit IgG Alexa-Fluor 488 (1:1,000; Cat. No. A21206; Life Technologies Co. Eugene, OR, United States) and anti-mouse IgG Alexa-Fluor 568 (1:1,000; Cat. No. A11004; Life Technologies Co., Eugene, OR, United States). Following secondary incubation, sections were washed five times in 0.1 M PB, mounted onto glass slides, cover-slipped using *Fluoroshield Mounting Medium*, and sealed with nail polish, once cured. Each slide was mounted with brain slices from one subject. The images from each slide (three slices *per* brain area) were acquired using a fluorescence microscope (Axio Imager.D2, Carl Zeiss Microscopy, LLC, Thornwood, NY, United States)-connected Zen Pro 2.0 software (Carl Zeiss Microscopy, LLC, Thornwood, NY, United States) and were analyzed using ImageJ software (NIH). The corrected total cellular fluorescence [CTCF = integrated density – (area of selected tissue area × mean fluorescence of background readings)] of the RmPFC was measured by subtracting the background fluorescence from the integrated intensity and performed as described previously (Burgess et al., 2010; McCloy et al., 2014; Baptista-de-Souza et al., 2020). Thus, the final CTCF was the average of three slices/brain subareas/mouse from six to seven mice/group.

### General Procedure

#### Experiment 1: Behavioral Effects of Chronic Social Defeat Stress in Mice Tested on Social Interaction Test

Twenty-four mice were subjected to 10 SDS (*n* = 15) or non-aggressive interactions (*n* = 9) (see the section “Social Defeat Stress” for details), and 24 h later, they were exposed to the social interaction test (SIT) for social avoidance behavior assessment.

#### Experiment 2: Behavioral Effects of Chronic Social Defeat Stress in Mice Exposed to the Elevated Plus Maze

Seventeen mice were subjected to 10 SDS (*n* = 10) or non-aggressive interactions (*n* = 7) (see the section “Social Defeat Stress” for details), and 24 h later, they were exposed to the EPM.

#### Experiment 3: Effects of Chronic Social Defeat Stress on ΔFosB, CaMKII, and ΔFosB + CaMKII Labeling in the Medial Prefrontal Cortex

Thirteen mice were subjected to 10 sessions of SDS (*n* = 7) or non-aggressive interaction (*n* = 6) (see section the “Social Defeat Stress” for details), and 24 h later, they were euthanized, and their brains were removed to immunofluorescence assay to identify ΔFosB, CaMKII, and ΔFosB + CaMKII-labeled neurons in the mPFC. An experimentally naïve group (*n* = 6) was also added to this experiment to record the basal levels of immunofluorescence.

#### Experiment 4: Evidence of Projections From the Left Prelimbic to the Right Medial Prefrontal Cortex

Five mice received neurotracer microinfusion within the left PrL area (see the section “Surgery and Neurotracer and Drug Microinjection” for details), and 3 weeks later, they were euthanized, and their brains were removed and histologically processed for the verification of projection analyses.

#### Experiment 5: Effects of Intra-prelimbic Injection of Cobalt Chloride on Anxiety, cFos, CaMKII, and cFos + CaMKII Labeling in the Right Medial Prefrontal Cortex

Six days after surgery, 25 mice were transported to the experimental room and left undisturbed for at least 30 min before testing. Saline or CoCl_2_ (1.0 mM/0.2 μl) was injected into the PrL area of the left mPFC and, 10 min later, each animal was placed on the EPM to record the anxiety (%OE and %OT) and locomotion (CE) indices for a 5-min period. A 90 min later, the animals were euthanized, and their brains were removed for immunofluorescence assay to identify cFos, CaMKII, and cFos + CaMKII-labeled neurons in the RmPFC.

#### Experiment 6: Effects of 2-Amino-7phosphonoheptanoic Acid and Cobalt Chloride Injected Into the Right Dorsal Medial Prefrontal Cortex and Left Dorsal Medial Prefrontal Cortex, Respectively, on the Anxiety of Mice Exposed to the Elevated Plus Maze

Six days after surgery, 78 mice were transported to the experimental room and left undisturbed for at least 30 min before testing. Then, saline or AP7 (0.05 nmol/0.2 μl) was injected into the right dorsal mPFC (Cg1 + PrL) followed by saline or CoCl_2_ (1.0 mM/0.2 μl) injection into the left dorsal mPFC and, 10 min later, each mouse was exposed to the EPM to record the anxiety indices (%open-arm entries and %open-arm time) and locomotion (closed-arm entries) for a 5-min period.

### Statistical Analysis

All results were initially subjected to Levene’s test for homogeneity of variance. The data of experiments 2 and 5 were submitted to Student *t*-test for independent samples. Two-way analysis of variance (ANOVA) was used to evaluate the data from experiment 1 [factor 1: condition (NA or Stress); factor 2: target (no target or target)], and experiment 6 [factor 1: treatment in the LmPFC (saline or CoCl_2_); factor 2: treatment in the RmPFC (saline or AP7)]. The data of experiment 3 were submitted to the two-way ANOVA for repeated measures [factor 1: condition (Naïve, NA, or Stress); factor 2: side (left or right)]. When significant, data were further analyzed using the Tukey–Kramer *post hoc* test. Values of p ≤ 0.05 were considered significant.

## Results

### Experiment 1: Chronic Social Defeat Stress Increases Social Avoidance Behavior

**FIGURE 1:**
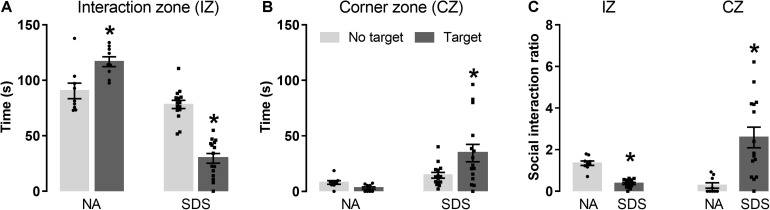
Chronic SDS induces social avoidance-like behavior in mice. **(A)** SDS mice (*n* = 15) spend less time in the interaction zone (IZ) with a nonfamiliar target compared with the NA interaction mice (*n* = 9). **(B)** Time spent in the corner zone (CZ) in the absence and presence of the target. **(C)** Social interaction ratio in the IZ and CZ. Bars with scatter dot plot represent mean (±SEM). *p < 0.05 compared with the No target of the respective group or to the NA group. NA, non-aggressive; SDS, social defeat stress.

Figures 1A,B represent the time spent in the (Figure 1A) interaction zone (IZ) or (Figure 1B) corner zone (CZ) exhibited by non-aggressive (NA; *n* = 9) and chronic SDS mice (SDS; *n* = 15) in the absence (no target) and presence (target) of a resident conspecific. A two-way ANOVA for repeated measures indicated significative effects for the exploration time in the IZ for condition [*F*(1,22) = 89.63; *p* < 0.05] and target [*F*(1,22) = 5.98; *p* < 0.05] factors as well as for between-factor interaction [*F*(1,22) = 68.43; *p* < 0.05]. *Post hoc* test revealed that the NA mice explored more the IZ in the presence than in the absence of the target. In contrast, SDS mice spent less time in the IZ in the presence than in the absence of the target (Figure 1A). A two-way ANOVA for repeated measures also indicated significant changes in the CZ for condition [*F*(1,22) = 8.97; *p* < 0.05] and target [*F*(1,22) = 3.11; *p* < 0.05] factors as well as for between-factors interaction [*F*(1,22) = 8.66; *p* < 0.05]. *Post hoc* test revealed that the SDS group also spent more time in the CZ with the target compared with the situation wherein the target is not present (Figure 1B).

Figure 1C represents the social interaction ratio in the IZ and CZ of NA and SDS mice. Student’s *t*-test indicated a decrease in the IZ [*t*_(22)_ = 9.36, *p* < 0.05] and an increase in the CZ [*t*_(22)_ = –3.53, *p* < 0.05]. These results demonstrate an increase in the social avoidance behavior in the SDS group compared with the NA group (Figure 1C).

### Experiment 2: Anxiety-Like Behavior Induced by Chronic Social Defeat Stress

**FIGURE 2:**
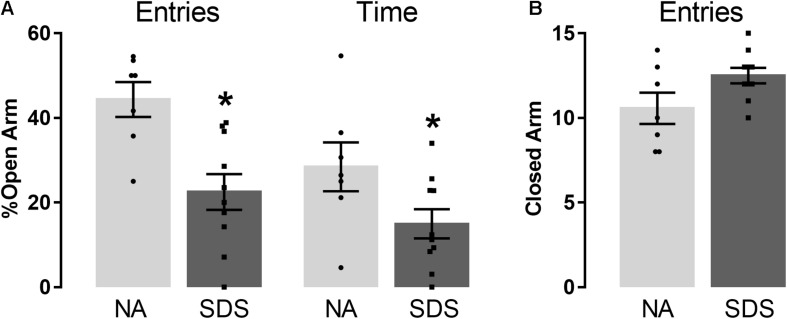
Chronic social defeat stress induces anxiety-like behavior in mice. **(A)** percentage of open-arm entries and percentage of open-arm time in the EPM. **(B)** Frequency of closed-arm entries in the EPM. Bars with scatter dot plot represent mean (±SEM). *n* = 7–10. *p < 0.05 compared with the NA group. NA, non-aggressive; SDS, social defeat stress; EPM, elevated plus maze.

Figure 2 represents the percentage of entries and time in the open arm (Figure 2A), and the frequency of closed arm entries (Figure 2B) of mice subjected to non-aggressive or chronic SDS interaction and exposed to the EPM. Student’s *t*-test indicated that SDS animals (*n* = 10) reduced the percentage of open-arm exploration (entries and time) compared with NA animals (*n* = 7) [entries (*t*_(15)_ = 3.58; *p* < 0.05) and time (*t*_(15)_ = 2.14; *p* < 0.05)] (Figure 2A). No significant differences were recorded and found for the number of closed-arm entries [*t*_(15)_ = 2.06; *p* > 0.05] (Figure 2B). These results demonstrate an increase in anxiety-like behavior in the SDS group compared with the NA group.

### Experiment 3: Chronic Social Defeat Stress Promotes Differential Activation Pattern in ΔFosB, CaMKII, and ΔFosB + CaMKII Labeling in the Medial Prefrontal Cortex Subareas (Cingulate, Prelimbic, and Infralimbic)

**FIGURE 3:**
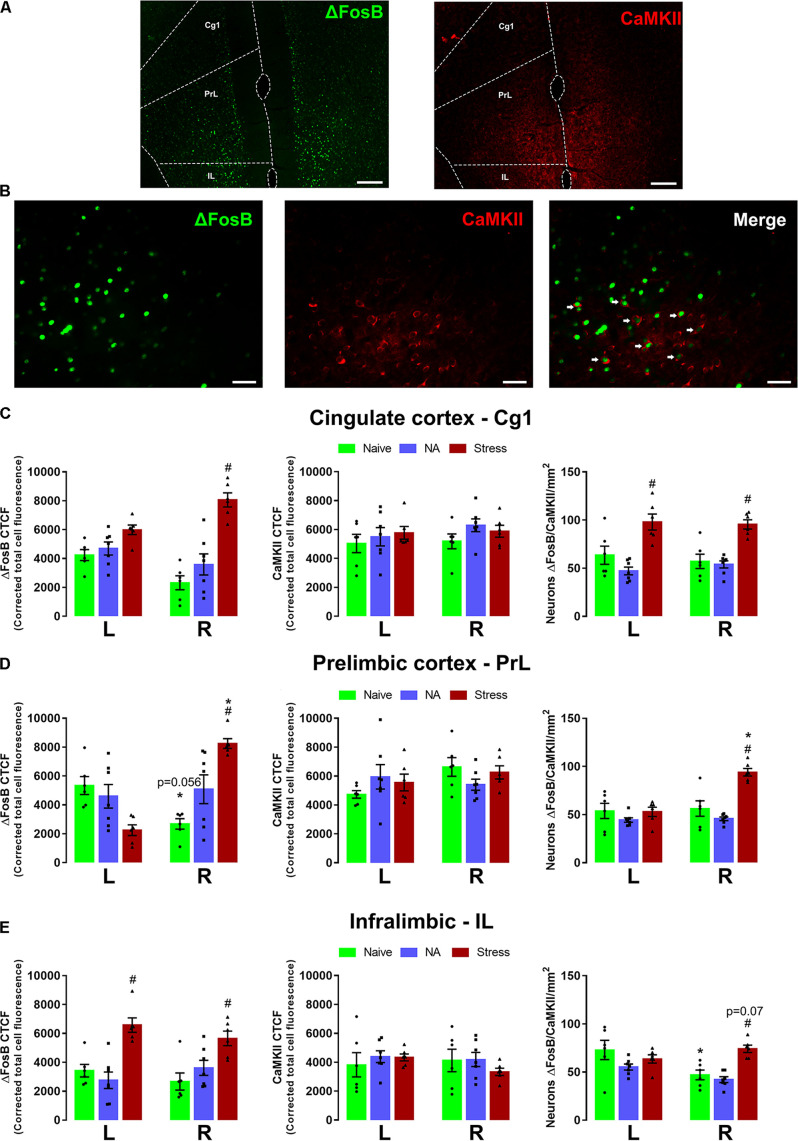
Representative × 10 (scale bar = 200 μm) **(A)** and × 40 (scale bar = 20 μm) **(B)** images showing ΔFosB, CaMKII immunoreactivity, and double-labeling merge for ΔFosB and CaMKII in the mPFC. Corrected total cell fluorescence (CTCF) for ΔFosB and, CaMKII, and ΔFosB + CaMKII-positive neurons in the Cg1 **(C)**, PrL **(D),** and IL **(E)**. Sample sizes: Naive (*n* = 6), NA (*n* = 7), and SDS (*n* = 6). Bars with scatter dot plot represent mean (±SEM). *p < 0.05 compared with the same group on the opposite hemisphere. ^#^*p* < 0.05 compared with the naive and NA group. L, left hemisphere; R, right hemisphere; NA, non-aggressive; SDS, social defeat stress; mPFC, medial prefrontal cortex; Cg1, cingulate; PrL, prelimbic; IL, intralimbic.

Figure 3 represents the activation pattern in ΔFosB, CaMKII, and ΔFosB + CaMKII (merge) labeling in Cg1, PrL, and IL subareas of the left and right mPFC. The brain of experimentally naïve (*n* = 6) mice and those exposed to non-aggressive (*n* = 7) or SDS (*n* = 6) interaction were subjected to immunofluorescence assay.

#### Cg1

Two-way ANOVA for repeated measures of the cingulate cortex **(Cg1 area)** indicated significant differences on ΔFosB labeling for stress condition factor [*F*(2,16) = 31.12; *p* < 0.05], and for condition versus side interaction [*F*(2,16) = 7.46; *p* < 0.05], but not differences for side factor [*F*(1,16) = 0.54; *p* > 0.05]. *Post hoc* test showed a higher ΔFosB expression in the right Cg1 of the SDS group compared with the respective naïve mice. For CaMKII labeling, two-way ANOVA for repeated measures did not indicate any significant effect [condition: *F*(2,16) = 1.72; *p* > 0.05; side: *F*(1,16) = 0.55; *p* > 0.05; condition × side interaction: *F*(2,16) = 0.23; *p* > 0.05]. For double-labeling (ΔFosB + CaMKII) analysis, two-way ANOVA for repeated measures indicated significant differences only for condition factor [*F*(2,16) = 21.88; *p* < 0.05; side: *F*(1,16) = 0.02; *p* > 0.05; condition × side interaction: *F*(2,16) = 0.81; *p* > 0.05]. *Post hoc* test revealed higher levels of ΔFosB + CaMKII labeling in both sides of stressed animals compared with their respective naïve and NA groups (Figure 3C).

#### PrL

Two-way ANOVA for repeated measures for ΔFosB labeling indicated significant differences on ΔFosB labeling for side factor [*F*(1,16) = 7.20; *p* < 0.05] and for condition versus side interaction [*F*(2,16) = 27.09; *p* < 0.05], but not differences for condition factor [*F*(2,16) = 1.31; *p* > 0.05]. *Post hoc* test revealed a trend of lower ΔFosB labeling in the right compared with the left hemisphere of naïve mice (*p* = 0.056). ΔFosB labeling in stressed animals was higher in the right side than in the left side and in the naïve group. Two-way ANOVA for repeated measures did not reveal any significant main factor effect for CaMKII labeling [condition: *F*(2,16) = 0.10; *p* > 0.05; side: *F*(1,16) = 2.28; *p* > 0.05; condition × side interaction: *F*(2,16) = 2.49; *p* > 0.05]. For double-labeling (ΔFosB + CaMKII) analysis, two-way ANOVA for repeated measures indicated significative main effect for all factors [condition: *F*(2,16) = 9.74; *p* < 0.05; side: *F*(1,16) = 34.60; *p* < 0.05; condition × side interaction: *F*(2,16) = 25.72; *p* < 0.05]. *Post hoc* test revealed an increase in the ΔFosB + CaMKII labeling only in the right side of the SDS group compared with the respective naïve and NA groups (Figure 3D).

#### IL

Two-way ANOVA for repeated measures indicated significant differences of ΔFosB labeling only for condition factor [*F*(2,16) = 26.06; *p* < 0.05; side: *F*(1,16) = 0.33; *p* > 0.05; condition × side: *F*(2,16) = 1.53; *p* > 0.05]. A *post hoc* test revealed that SDS animals presented higher ΔFosB labeling in both hemispheres than naïve and NA groups. Two-way ANOVA for repeated measures did not reveal any significant main factor effect for CaMKII labeling [condition: *F*(2,16) = 0.52; *p* > 0.05; side: *F*(1,16) = 0.35; *p* > 0.05; condition × side interaction: *F*(2,16) = 0.53; *p* > 0.05]. For ΔFosB + CaMKII double labeling, two-way ANOVA for repeated measures indicated significant effects for all three factors [condition: *F*(2,16) = 5.53; *p* < 0.05; side: *F*(1,16) = 7.92; *p* < 0.05; condition × side interaction: *F*(2,16) = 9.53; *p* < 0.05]. *Post hoc* test revealed a reduction in double labeling in the right compared with the left side in naïve animals. A trend of increase in double labeling was recorded in the right side of SDS mice compared with the respective naïve and NA groups (*p* = 0.07) (Figure 3E).

Photomicrographs illustrating immunofluorescence slices of ΔFosB, CaMKII, and ΔFosB + CaMKII labeling are shown in Figures 3A,B.

### Experiment 4: The Left Prelimbic Projects to the Right Medial Prefrontal Cortex

To identify potential direct neuronal projections from the left PrL to the right mPFC, we injected the fluorescent BDA marker, an anterograde tracer, into the left PrL (Supplementary Figure 1A) of five mice. Supplementary Figure 1B shows anterogradely labeled axons in the right mPFC visualized 3 weeks after BDA injection.

### The Anxiogenic Effect Produced by Cobalt Chloride Injected Into the Left Prelimbic Increases cFos and cFos + CaMKII Labeling in Neurons of the Right Prelimbic and Infralimbic Subregions

#### Histology

Supplementary Figure 2A shows a representative photomicrograph of the microinfusion site within the left PrL of the mouse. Furthermore, the mPFC can be subdivided into cingulate (Cg1), prelimbic (PrL), and infralimbic (IL) cortices (Paxinos and Franklin, 2001). To confirm the injection site, 0.1-μl solution of 2% Evans blue was microinjected into the mPFC before the perfusion procedure (section “Immunofluorescence”). Histology confirmed that a total of 19 mice had accurate cannula placement in the left PrL. Reaches outside of the PrL portion were excluded from the study.

**FIGURE 4:**
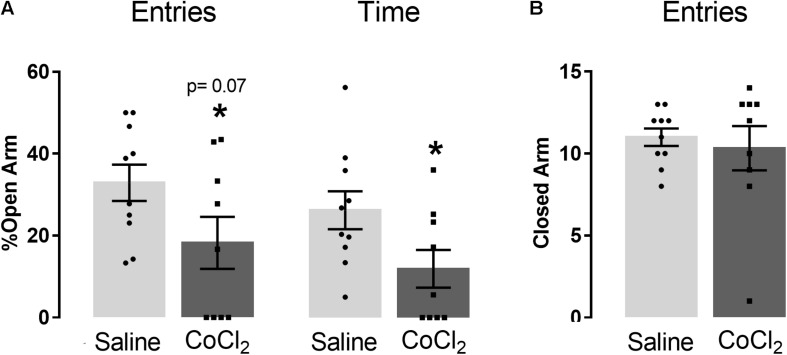
Anxiogenesis induced by left PrL inhibition (with local injection of CoCl_2_). **(A)** Effects of CoCl_2_ injection into the left PrL on the percentage of open-arm entries and percentage of open-arm time, and **(B)** frequency of closed-arm entries in the EPM of saline (*n* = 10) and CoCl_2_ (*n* = 9) groups. Bars with scatter dot plot represent mean (±SEM). *p < 0.05 compared with the saline group. CoCl_2_, cobalt chloride.

Student’s *t*-test indicated that mice treated with CoCl_2_ (*n* = 9) reduced the open-arm exploration compared with saline-treated animals (*n* = 10) [%entries (*t*_(17)_ = 1.93; *p* = 0.07) and %time (*t*_(17)_ = 2.18; *p* < 0.05)] (Figure 4A). No significant between-group differences were indicated in the number of closed-arm entries (*t*_(17)_ = 0.48; *p* > 0.05) (Figure 4B).

**FIGURE 5:**
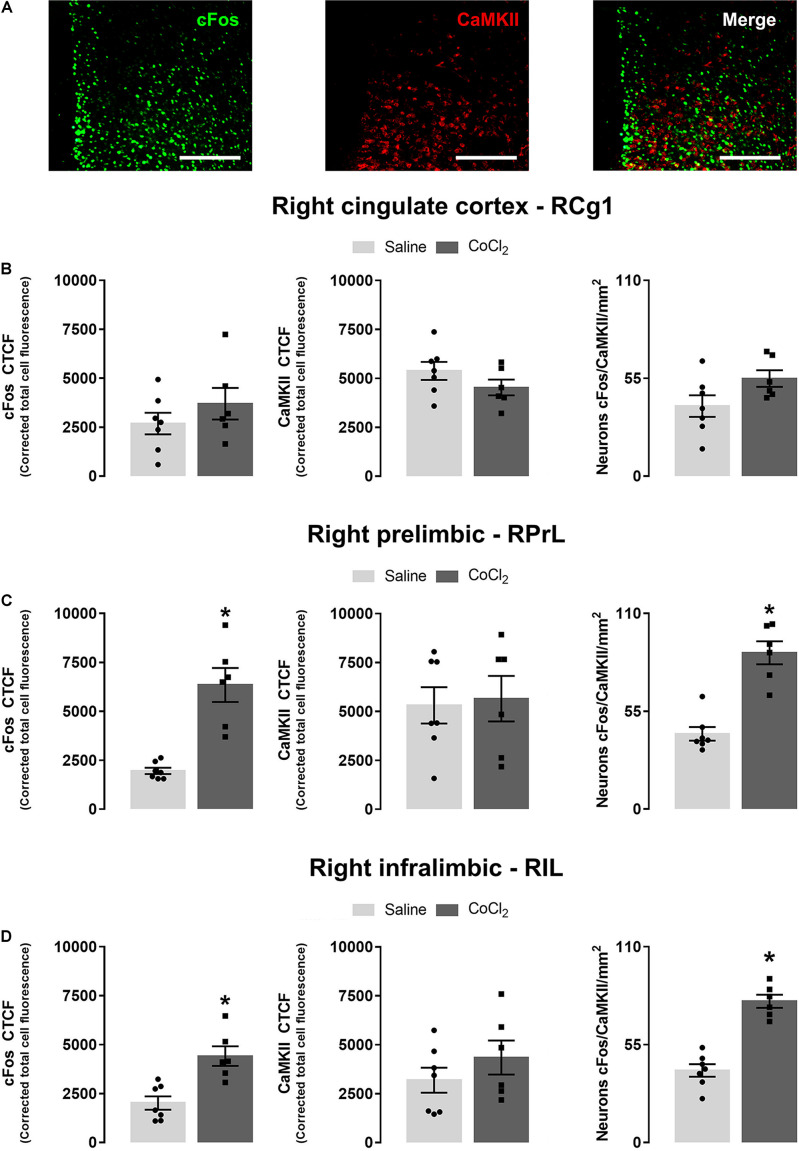
Anxiogenesis induced by left PrL inhibition (with local injection of CoCl_2_) increases cFos and cFos + CaMKII labeling in the right PrL and IL (but not Cg1) subareas. **(A)** Representative × 20 images showing cFos, CaMKII, and double labeling for cFos and CaMKII on the right mPFC (scale bar = 100 μm). Corrected total cell fluorescence (CTCF) for cFos, CaMKII, and cFos + CaMKII-positive neurons in the RCg1 **(B)**, RPrL **(C),** and RIL **(D)**. Saline (*n* = 7) and CoCl_2_ (*n* = 6). Bars with scatter dot plot represent mean (±SEM). *p < 0.05 compared with the saline group. CoCl_2_, cobalt chloride.

For immunofluorescence assay analysis, data from the right mPFC of seven mice from the CoCl_2_ group and six animals from the saline group were evaluated. Student’s *t*-test indicated no significant between-group differences in the right *Cg1* for cFos [*t*_(__11)_ = 1.061; *p* > 0.05], CaMKII [*t*_(__11)_ = 0.97; *p* > 0.05] and for cFos + CaMKII colocalization [*t*_(__11)_ = –1.97; *p* > 0.05] (Figure 5B). For *PrL* subregion, Student’s *t*-test indicated that the injection of CoCl_2_ in the left PrL increased cFos [*t*_(__11)_ = –5.39; *p* < 0.05] and cFos + CaMKII labeling in neurons of right PrL portion [*t*_(__11)_ = –6.32; *p* < 0.05]. The analysis for CaMKII-positive neurons did not indicate significant differences between groups [*t*_(11__)_ = –0.23; *p* > 0.05] (Figure 5C). Finally, Student’s *t*-test indicated that the injection of CoCl_2_ into the left *PrL* also increased cFos [*t*_(__11)_ = –4.06; *p* < 0.05] and cFos + CaMKII [*t*_(__11)_ = –7.63; *p* < 0.05], without changing CaMKII [*t*_(__11)_ = –1.10; *p* > 0.05] labeling in the IL portion of the RmPFC (Figure 5D). Photomicrographs illustrating immunofluorescence slices of cFos, CaMKII, and cFos + CaMKII labeling are shown in Figure 5A.

#### Experiment 6: Blockade of NMDA Receptor in the Right Prelimbic Impairs the Anxiogenic-Like Effects Produced by Injection of Cobalt Chloride Into the Left Prelimbic

##### Histology

Supplementary Figure 2B shows a representative photomicrograph of the microinfusion site within the left and right PrL of the mouse. To confirm the injection site, 0.1-μl solution of 2% Evans blue was microinjected into the PrL before the perfusion procedure (section “Immunofluorescence”). Histology confirmed that a total of 33 mice had accurate bilateral cannula placement in the PrL. Although our target site has been the PrL, a total of 27 mice had bilateral cannula placement in the Cg1 and were included in the final analysis. The off-target microinfusion sites (e.g., IL) that were excluded from the final analysis are represented as a photomicrograph in Supplementary Figure 2C.

##### Only injections into the Cg1

**FIGURE 6:**
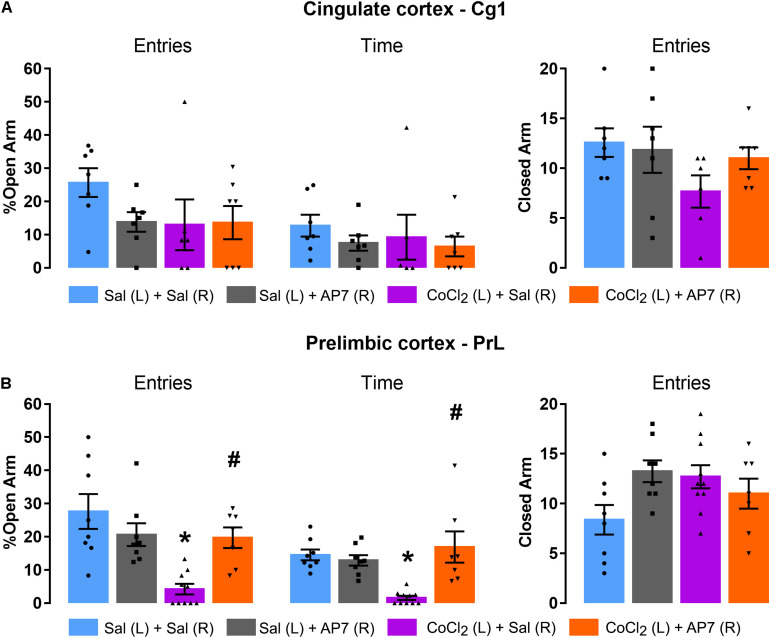
Effects of AP7 injected into the right dorsal mPFC (R) subsequently to CoCl_2_ injection into the left dorsal mPFC (L) on behavior of mice exposed to the EPM. **(A)** Cg1 analysis on the percentage of open-arm entries and time, and frequency of closed-arm entries [Sal (L) + Sal (R) (*n* = 7); Sal (L) + AP7 (R) (*n* = 7); CoCl_2_ (L) + Sal (R) (*n* = 6); CoCl_2_ (L) + AP7 (R) (*n* = 7)]. **(B)** PrL analysis of the percentage of open-arm entries and percentage of open-arm time, and frequency of closed-arm entries [Sal (L) + Sal (R) (*n* = 8); Sal (L) + AP7 (R) (*n* = 8); CoCl_2_ (L) + Sal (R) (*n* = 10); CoCl_2_ (L) + AP7 (R) (*n* = 7)]. Bars with scatter dot plot represent mean (±SEM). *p < 0.05 compared with Sal (L) + Sal (R) group; ^#^*p* < 0.05 compared with CoCl_2_ (L) + Sal (R) group. L, left; R, right; Sal, saline; AP7, 2-amino-7-phosphonoheptanoic acid—an NMDA glutamate receptor antagonist; CoCl_2_, cobalt chloride.

Figure 6A represents the lack of effects of CoCl_2_ (saline or CoCl_2_; factor 1) and AP7 (saline or AP7; factor 2) injected into the left Cg1 and right Cg1 subregions, respectively, on the anxiety-like behavior of mice exposed to the EPM. Two-way ANOVA indicated no significant changes in open-arm exploration [% entries: factor 1 *F*(1,23) = 1.63; *p* > 0.05, factor 2 *F*(1,23) = 1.23; *p* > 0.05, and factor 1 × factor 2 factor interaction *F*(1,23) = 1.54; *p* > 0.05; %time: factor 1 *F*(1,23) = 0.32; *p* > 0.05, factor 2 *F*(1,23) = 1.03; *p* > 0.05; factor 1 × factor 2 interaction *F*(1,23) = 0.09; *p* > 0.05] and closed-arm entries [factor 1 *F*(1,23) = 2.92; *p* > 0.05, factor 2 *F*(1,23) = 0.60; *p* > 0.05, and factor 1x factor 2 factor interaction *F*(1,23) = 1.44; *p* > 0.05].

##### Only injections into the PrL

Figure 6B represents the effects of CoCl_2_ (saline or CoCl_2_; factor 1) and AP7 (saline or AP7; factor 2) injected into the left PrL and right PrL subregion, respectively, on the anxiety-like behavior of mice exposed to the EPM. Two-way ANOVA indicated significant changes in the exploration of the open arms of mice treated with saline or CoCl_2_ into the left PrL and saline or AP7 into the right PrL [%entries: factor 1: *F*(1,29) = 12.32; *p* < 0.05; factor 2: *F*(1,29) = 1.51; *p* > 0.05; factor 1 × factor 2 interaction *F*(1,29) = 10.50; *p* < 0.05; %time: factor 1 *F*(1,29) = 3.79; *p* > 0.05, factor 2: *F*(1,29) = 8.80; *p* < 0.05; and factor 1 × factor 2 interaction: *F*(1,29) = 13.58; *p* < 0.05]. *Post hoc* test revealed that CoCl_2_ injection reduced both percentage of entries and percentage of time in open arms only in those animals that had received saline into the right PrL. In other words, the injection of AP7 into the right PrL subregion of the mPFC impaired the anxiogenic-like effects produced by the left PrL inhibition. Two-way ANOVA for frequency of closed-arm entries did not reveal significant effects for factor 1 [*F*(1,29) = 0.62; *p* > 0.05] and factor 2 [*F*(1,29) = 1.45; *p* > 0.05], but showed significant effects for factor 1 × factor 2 interaction [*F*(1,29) = 6.21; *p* < 0.05]. Tukey–Kramer *post hoc* test revealed no significant difference compared with the saline + saline group.

## Discussion

The behavioral results have demonstrated that chronic SDS increases social avoidance/anxiogenic-like behaviors in mice. In the social interaction test, chronic SDS induced social avoidance behavior. Furthermore, the results indicated that this protocol promoted an expressive decrease in the open-arm exploration in the EPM, suggesting the anxiogenic-like profile in stressed animals.

The SDS procedure was based on the protocol described by Golden et al. (2011), wherein the authors have demonstrated that 10 repeated agonistic social confrontations provoke a decrease in the social interaction in the defeated animal. The results from the social interaction and EPM tests corroborate previous studies demonstrating the emotional-like consequence induced by SDS (Warren et al., 2013, 2014; Carnevali et al., 2020). It is important to highlight that these emotional chains of reactions can be attenuated by anxiolytics and antidepressant drugs, like diazepam, venlafaxine, tianeptine, and reboxetine (Rygula et al., 2008; Venzala et al., 2012; Lkhagvasuren et al., 2014).

Previous findings have demonstrated the distinct modulation of the mPFC hemispheres in the sustained stress animal models (Sullivan and Gratton, 1999; Radley et al., 2006; Cerqueira et al., 2008; Lee et al., 2015; Costa et al., 2016; Faria et al., 2020; Victoriano et al., 2020). Here, the immunofluorescence results demonstrated differential changes in the ΔFosB expression (a sustained neuronal activity marker) (Perrotti et al., 2004; Nestler, 2013) and double labeling of ΔFosB/CaMKII (the downstream factor following NMDA receptor activation), depending on the mPFC subarea and hemispheric side. For instance, in the Cg1 area, chronic SDS induced a rise in the ΔFosB expression in the right side and bilateral rise in the ΔFosB/CaMKII double labeling, whereas in the PrL, stressed animals presented an increase in the right hemisphere of ΔFosB. Similar lateralization was observed for ΔFosB + CaMKII neurons wherein the increase was detected only on the right side. In the IL, chronic SDS promoted a higher ΔFosB expression bilaterally and produced a tendency to increase the double labeling on the right side.

Interestingly, in naïve mice, analyses of the PrL mPFC pointed out a borderline effect (*p* = 0.056) showing a lower expression of ΔFosB in the right hemisphere than in the left hemisphere. This finding seems to corroborate previous studies suggesting that, in basal conditions, the left mPFC would inhibit the right mPFC (Sullivan, 2004; Cerqueira et al., 2008). If so, present results suggest that this modulation could occur only in the PrL subarea. Furthermore, this reduction was not present in the CaMKII and double-labeled neurons located in the RmPFC, suggesting that the potential inhibition exerted by the LmPFC over the RmPFC would not depend on the glutamatergic neurons or would involve the activation of gabaergic interneurons in the right hemisphere. We will come back again across this assumption below. On the other hand, in those cases where the immunofluorescence analyses were carried out specifically in the PrL, chronic SDS produced a bidirectional profile on ΔFosB expression in the left and right hemispheres, i.e., while a tendency to decrease in ΔFosB labeling was observed in the left side, a marked increase in this neuronal marker was detected in the right PrL in SDS mice. This effect suggests that chronic SDS events would lead to a loss of control of the left side over the right side, which, in turn, becomes more active and recruits glutamatergic neurotransmission as shown by the increase in ΔFosB + CaMKII double labeling in this hemisphere. Present results corroborate previous studies demonstrating the predominant role of the right mPFC on sustained SDS (Faria et al., 2020; Victoriano et al., 2020) and suggest that the right PrL (but not the Cg1 and IL) subarea is particularly sensitive to the reduction in the left PrL ΔFosB labeling induced by chronic SDS. In this context, previous studies have demonstrated that impairments in the neuronal excitability of mPFC provoked by chronic stress may cause morphological alteration in this area (Radley et al., 2004, 2006, 2008; Gilabert-Juan et al., 2013; McKlveen et al., 2016). Considering that the present results have mostly indicated that the increase in neuronal activity (i.e., raised ΔFosB) was followed by the accentuation of ΔFosB + CaMKII double labeling, we suggest that the glutamatergic neurons located in the right mPFC play an important role in the modulation of the emotional consequences induced by chronic SDS. Although attractive, these assumptions need to be clarified in further studies using more advanced techniques.

Previous studies have demonstrated that the mPFC projects to several limbic areas (e.g., amygdala, hippocampus, hypothalamus, and BNST) that modulate emotional responses (Gabbott et al., 2005; Cerqueira et al., 2008; Kim and Cho, 2017; Ko, 2017; Giannotti et al., 2019). In addition, there is dense intracortical connectivity, indicating subregion integration (McKlveen et al., 2019). On this wise, Marek et al. (2018) have demonstrated, using retro-beads and Fos expression techniques, a unidirectional excitatory connection from the PrL to the IL in a fear extinction model. Although in that elegant study the authors did not specify or discuss their results concerning functional laterality, it is reasonable to suggest, considering the findings from our research group (Costa et al., 2016; Faria et al., 2020; Victoriano et al., 2020) and the qualitative results shown in the present study (Supplementary Figure 1A) that the left PrL projects to the right side. If so, we suggest that the PrL neurons located in the left mPFC may play a direct or even indirect (via subcortical areas; e.g., amygdala, BNST) role in the right mPFC in the modulation of the avoidance behavior provoked by a stressful situation. However, we are aware that the results that are shown in experiment 4 (Supplementary Figure 1A) are limited to a qualitative observation. Thus, further studies using more advanced tools are necessary to clarify whether the LmPFC projects directly or indirectly to the RmPFC.

To investigate this functional lateralization of the PrL, we demonstrated that synaptic inactivation of the left PrL through local injection of CoCl_2_, an unspecific synaptic inhibitor (Kretz, 1984), produced anxiogenesis (experiments 5 and 6), which is abolished when the NMDA receptors located in the right PrL are blocked with local injection of AP7 (experiment 6). Interestingly, CoCl_2_ injection into the left PrL also increases cFos + CaMKII double labeling in the contralateral PrL and IL portions, suggesting that the anxiogenesis induced by the inhibition of the left PrL leads to glutamate NMDA receptor activation in the right PrL and IL subareas. These results suggest that the left PrL inactivation triggers glutamate releasing in the RmPFC, which, in turn, provokes anxiogenesis via NMDA receptor activation. Besides the anxiogenic-like effect, left PrL inhibition induced an increase in the cFos expression in neurons of the PrL and IL (but not in the Cg1) of the RmPFC. Taken together, these results show an important interaction between mPFC hemispheres in the modulation of anxiety, wherein the left activity seems to modulate the level of activation of the RmPFC, notably via NMDA glutamate receptors.

While the anxiogenic-like effect induced by intra-LmPFC injection of CoCl_2_ has already been demonstrated in mice exposed to the EPM (Costa et al., 2016; Victoriano et al., 2020), the present work brings more accurate data regarding the mPFC subareas. It is important to highlight that the initial aim of this study was to investigate the role of the dorsal region of the mPFC (i.e., Cg1 and PrL) in the modulation of avoidance/anxiety responses induced by local injection of CoCl_2_ in the left hemisphere. However, after analyzing the data, we detected differences in the behavioral pattern when the cannulation reached these specific subareas, and therefore, we sought to investigate the role of the Cg1 and PrL subregions separately. As a consequence, a clear anxiogenic-like effect was observed with CoCl_2_ injection into the left PrL, but not into the Cg1, suggesting a marked role of this subarea in the modulation of anxiety. Interestingly, such effect disappeared when the contralateral PrL received the NMDA receptor blocker AP7. In such conditions, these animals (CoCl_2_ + AP7) explored the aversive area of the EPM similarly to those treated with saline (saline + saline or saline + AP7). It is important to notice that the used dose of AP7 (0.05 nmol) did not change *per se* the anxiety-like measures. Moreover, synaptic inhibition of the left PrL or NMDA blockade of the right PrL did not change the frequency of closed-arm entries, a widely used measure of general activity (Cruz et al., 1994; Rodgers and Johnson, 1995), suggesting that the drug effects were selective on anxiety indices. Taken together, the present results indicate that mPFC subareas do not play a similar role in the modulation of the anxiety of mice exposed to the EPM. Thus, grouping mPFC subregions as one may lead to misinterpretation of an acquired data.

The mPFC is composed of 80–90% of glutamatergic neurons, which are under inhibitory regulation by interneurons, being most of them GABAergic neurons (Harris and Shepherd, 2015; McKlveen et al., 2015). Through an intricate pathway, the flow of local information within the mPFC (that is sent to subcortical structures afterward) is under complex functional control. In this sense, Cerqueira et al. (2008) have postulated that, in basal conditions, the RmPFC would be under tonic inhibition from its left counterpart, and, after chronic stress situations, there would be a disruption on this control, notably by left dendritic arborization loss (Czéh et al., 2007) (leading to a reduction in the neuronal excitability and synaptic plasticity). The reduced function of the LmPFC would lead to augmented activity of the right side, facilitating hormonal stress response, through the HPA (hypothalamic–pituitary–adrenal) axis stimulation. Also, in this scenario, mPFC–amygdala inhibition is decreased, whereas the BNST and sympathetic tonus, are increased (Cerqueira et al., 2008). Our results strengthen this view about the functional lateralization of the mPFC since chronic social defeat stress altered the pattern of ΔFosB and ΔFosB + CaMKII double-labeling expression according to the mPFC subarea. Moreover, the left-side inhibition increased the right-side activation, assessed through cFos + CaMKII protein investigation and NMDA receptor blockade. Thus, the attenuation of the left PrL inhibition-induced anxiogenesis produced by injection of AP7 into the right PrL suggests that the NMDA glutamate receptor located in the RmPFC plays an important role in the mediation of anxiety responses. Again, although attractive, this hypothesis needs to be clarified by further experiments involving more advanced molecular and pharmacological approaches (e.g., use of DREADS to identify which neurons and neurotransmitters are markedly playing a role in the modulation of the SDS-induced anxiety). In this context, and considering the wide density of glutamatergic neurons within the mPFC (Stern et al., 2010; McKlveen et al., 2015; Carvajal et al., 2016), it would not be unreasonable to suggest that, in normal conditions, glutamate projections from the LmPFC might be stimulating inhibitory (e.g., GABA) interneurons in the RmPFC. GABA, in turn, would inhibit local excitatory neurons (e.g., glutamate, CRF, others?). This could explain why ΔFosB + CaMKII double labeling in the left and right PrL did not differ from each other in basal conditions (i.e., naïve and NA groups) (Figure 3D). Gaba would also inhibit excitatory output neurons (e.g., glutamate, CRF, others?) that project to subcortical (e.g., amygdala, BNST, and hypothalamus) and midbrain (e.g., periaqueductal gray) areas that play a marked role in the modulation of anxiety-related responses (McNaughton and Corr, 2004; Cerqueira et al., 2008; Cooper et al., 2015). If so, under chronic SDS conditions, the LmPFC would reduce glutamate release in the RmPFC leading to a disruption in the anxiety control. In favor of this assumption are recent findings showing that (i) injection of AP7 into the right PrL impairs the anxiogenesis induced by SDS (Victoriano et al., 2020) and (ii) intra-BNST injection of NMDA or CRF receptor antagonists attenuate the anxiogenic effect induced by nitrergic activation of the RmPFC or by chronic SDS in mice (Faria et al., 2020). Further studies are currently being conducted by our research group aiming to clarify these hypotheses.

Sanacora et al. (2012) suggested that various types of stress promote the release of glutamate in cortical and limbic areas in humans and animals, an effect that has been related to mood and anxiety disorders. Taking the present results together, we suggest that left PrL inhibition leads to right PrL disinhibition, (i.e., activation), via NMDA receptor modulation. Thus, the left PrL would project to the RmPFC activating interneurons, which would inhibit PrL, preventing exacerbated responses to aversive stimuli. If so, when the left PrL is inactivated (e.g., through CoCl_2_ injection), the following steps might occur: (1) The right inhibitory interneurons of the mPFC lose their tonus. (2) Glutamate release is increased in the RmPFC. (3) NMDA receptor activation leads to an increase in Fos and CaMKII expression, and as a consequence, mice exhibit anxiogenic-like behavior.

Finally, our study highlights (i) that the inhibition of left PrL might be used as a tool for inducing behavioral and functional alterations quite similar to those caused by chronic SDS and (ii) the importance of not considering the functional role of the mPFC as a unitary structure, whether it is related to the dorsoventral division or even to the hemispheric location.

## Conclusion

The present study demonstrates that (i) chronic SDS can induce social avoidance/anxiogenic-like behaviors and distinct neuronal activation/inhibition of the left and right mPFC subregions, and (ii) the left mPFC chemical inhibition also induces anxiety, an effect that is strongly related to the PrL subarea of the mPFC of mice. These findings and the evidence showing that the left mPFC projects directly to the right mPFC suggest that the left PrL modulates the neuronal activity of the right PrL and IL, whose stimulation elicits anxiety in mice exposed to the EPM. The left mPFC seems to play a tonic role in the modulation of anxiety, since its functional inhibition, particularly in the PrL subarea, led mice to avoid the open arms of the EPM. Together, the pharmacological manipulation (injection of AP7) added to the immunofluorescence analyses (Fos + CaMKII double labeling) show the crucial role of the glutamatergic system (through NMDA receptors) in the right PrL and IL subareas in the mediation of anxiogenesis in male mice.

## Data Availability Statement

The original contributions presented in the study are included in the article/Supplementary Material, further inquiries can be directed to the corresponding author/s.

## Ethics Statement

The animal study was reviewed and approved by the Ethical Committee for Use of Animals of the School of Pharmaceutical Sciences/Unesp, which complies with Brazilian and international guidelines for animal use and welfare (CEP/FCF/CAr-UNESP: protocol number 22/2017).

## Author Contributions

VF performed the first experiment. RN-D-S contributed to the conception and design of the study. All authors contributed to manuscript revision, read, and approved the submitted version.

## Conflict of Interest

The authors declare that the research was conducted in the absence of any commercial or financial relationships that could be construed as a potential conflict of interest.

## Publisher’s Note

All claims expressed in this article are solely those of the authors and do not necessarily represent those of their affiliated organizations, or those of the publisher, the editors and the reviewers. Any product that may be evaluated in this article, or claim that may be made by its manufacturer, is not guaranteed or endorsed by the publisher.

## Supplementary Material

Supplementary Figure 1 Photomicrographs depicting the anterogradely labeled neurons in the mPFC. **(A)** BDA microinjection site in the left PrL (scale bar = 100 μm). **(B)** White arrows show the presence of labeled neurons in the right PrL (scale bar = 50 μm). BDA (Dextran Amine-Texas Red^®^, Biotinylated), antegrade neurotracer.

Supplementary Figure 2 Representative photomicrographs of a representative microinfusion site. **(A)** Photomicrograph of a representative microinfusion site (black arrow) within the left prelimbic (Experiment 5). **(B)** Photomicrograph of a representative microinfusion site (black arrows) within the left and right prelimbic (Experiment 6). **(C)** Representative photomicrograph of the off-target microinfusion site (black arrows). Section corresponds to 1.94 mm anterior to bregma (Paxinos and Franklin, 2001). Scale bar = 1000 μm. Cg1, cingulate cortex, area 1; PrL, prelimbic cortex; IL, infralimbic cortex; fmi, forceps minor of the corpus callosum.
