# Supplementary figures and images for: Glutamatergic Neurotransmission Controls the Functional Lateralization of the mPFC in the Modulation of Anxiety Induced by Social Defeat Stress in Male Mice
Source: Front Behav Neurosci. 2021 Aug 23;15:695735. doi: 10.3389/fnbeh.2021.695735 (PMC8419264; doi:10.3389/fnbeh.2021.695735)

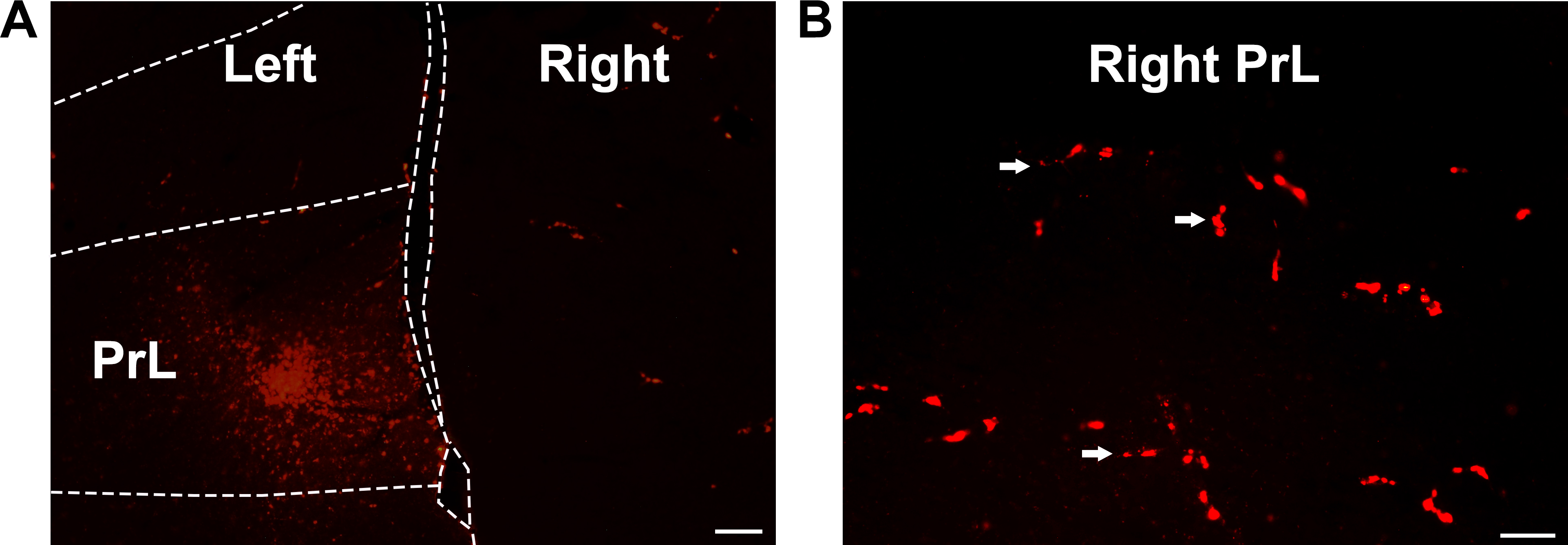

Supplement: Supplementary Figure 1 — Photomicrographs depicting the anterogradely labeled neurons in the mPFC. (A) BDA microinjection site in the left PrL (scale bar = 100 μm). (B) White arrows show the presence of labeled neurons in the right PrL (scale bar = 50 μm). BDA (Dextran Amine-Texas Red®, Biotinylated), antegrade neurotracer. [file Image_1.JPEG]
